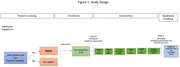# The Strengthening, engaging, and empowering dyads through nutrition & wellness study: a dyadic community‐based nutrition intervention for older adults with diabetes and cognitive impairment

**DOI:** 10.1002/alz.093149

**Published:** 2025-01-09

**Authors:** Mirnova E Ceide, Samantha Johnson, Ruchika Darapaneni, Jessica L Zwerling

**Affiliations:** ^1^ Montefiore Einstein, Bronx, NY USA

## Abstract

**Background:**

A meta‐analysis of over 2.3 million individuals in 14 studies showed that individuals with type II diabetes (T2DM) are at a 60% increased risk for development of any dementia compared to those without T2DM. A Whole Food, Plant Based dietary (WFPB) pattern has been associated with lower blood glucose levels and decreased insulin requirements. As older adults at risk for Alzheimer’s disease (AD) may be dependent on care partners for nourishment, it is imperative to involve the caregiving dyad in a lifestyle intervention. We propose a collaboration between our center of excellence for AD and a community‐based organization (CBO) that empowers people through WFPB nutrition. We will pilot a dyad‐focused nutritional educational series for older adults with T2DM and CI and their care partners in a local senior center.

**Method:**

In this single arm study, we will recruit 2 cohorts (English N = 15 and Spanish N = 15) of older adults (≥65) with T2DM and CI and their care partners to participate in 4 WFPB nutrition classes. Each class consists of: 1 hour of education and 1 hour of a culinary demonstration. We will also include 2 care partner support sessions co‐led by the nutrition facilitator and geriatric social worker at weeks 3 and 6.

**Result:**

In order to establish feasibility, we will assess important baseline characteristics of our participants including: food insecurity, cognition (Telephone MoCA), and multisensory integration (CatchU® mobility application). Acceptability, appropriateness, and feasibility of the intervention will be assessed quantitatively and qualitatively in debrief interviews. We will evaluate the preliminary efficacy on the intervention dietary pattern (including the MIND diet screen), diabetes self‐efficacy, and diabetes management: mean weekly glucose (logs and continuous glucose monitors).

**Conclusion:**

We will engage a high‐risk cohort of older adults with T2DM and CI through an inclusive and pragmatic study design. Rather than developing a novel nutrition intervention, we will leverage two local CBOs to provide the intervention and the location. This project will yield pilot data for a pragmatic randomized controlled trial of a dyadic nutrition intervention to improve metabolic health and cognition in older adults at‐risk for AD, especially those with diabetes.